# S-Adenosylmethionine regulates apoptosis and autophagy in MCF-7 breast cancer cells through the modulation of specific microRNAs

**DOI:** 10.1186/s12935-018-0697-6

**Published:** 2018-12-04

**Authors:** Concetta Paola Ilisso, Donatella Delle Cave, Laura Mosca, Martina Pagano, Alessandra Coppola, Luigi Mele, Michele Caraglia, Giovanna Cacciapuoti, Marina Porcelli

**Affiliations:** 1 Department of Precision Medicine, University of Campania “Luigi Vanvitelli”, Via L. De Crecchio 7, 80138 Naples, Italy; 2 Department of Experimental Medicine, University of Campania “Luigi Vanvitelli”, Naples, Italy

**Keywords:** S-Adenosylmethionine, Breast cancer cells, Apoptosis, Autophagy, MicroRNA

## Abstract

**Background:**

To get insight into the molecular mechanisms underlying the anti-tumor activity of S-adenosyl-l-methionine (AdoMet), we analyzed AdoMet-induced modulation of microRNAs (miRNAs) expression profile in MCF-7 breast cell line and its correlation with cancer-related biological pathways.

**Methods:**

MiRNA expression profiling was performed using a TaqMan MiRNA Array, following 500 µM AdoMet-treatment. The results were confirmed by Quantitative real-time PCR analysis. MCF-7 were transfected with miR-34a, miR-34c and miR-486-5p, mimics and inhibitors in presence or not of 500 µM AdoMet for 72 h. Apoptosis and autophagy were analyzed by flow cytometry and the modulation of the main antiproliferative signaling pathways were evaluated by Western blotting. The potential mRNA targets for each miRNA were identified by the TargetScan miRNA target prediction software.

**Results:**

Twenty-eight microRNAs resulted differentially expressed in AdoMet-treated MCF-7 cells compared to control cells. Among them, miRNA-34a and miRNA-34c were up-regulated while miRNA-486-5p was down-regulated. Moreover, we confirmed the ability of AdoMet to regulate these miRNAs in MDA-MB 231 breast cancer cell line. We demonstrate that, in MCF7 cells, the combination of either miR-34a or miR-34c mimic with AdoMet greatly potentiated the pro-apoptotic effect of AdoMet, by a caspase-dependent mechanism and activates p53 acetylation by inhibiting SIRT1 and HDAC1 expression. We also showed that miR-486-5p inhibitor induces autophagy and enhances AdoMet-induced autophagic process by increasing PTEN expression and by inhibiting AKT signaling.

**Conclusions:**

Our findings provide the first evidence that AdoMet can regulate miRNA expression in MCF-7 increasing our knowledge on the molecular basis of the antitumor effect of the sulfonium compound and suggest the use of AdoMet as an attractive miRNA-mediated chemopreventive and therapeutic strategy in breast cancer.

## Background

S-Adenosyl-l-methionine (AdoMet) is an important physiologic sulfonium compound that plays a primary role in cell metabolism as it represents the main methyl donor required in methylation reactions and the precursor of the decarboxylated S-adenosylmethionine, the propylamine group donor in polyamine biosynthesis [1–3]. AdoMet is biosynthesized from l-methionine and ATP by methionine adenosyltransferase (MAT, EC 2.5.1.6.) in a two-step reaction in which the energy-rich sulfonium compound is formed by the dephosphorylation of ATP [4–6]. All living cells express MAT highlighting its essential role in regulating appropriate levels of AdoMet [7].

Many scientific papers present in literature highlighted the ability of the AdoMet to inhibit tumor progression through the regulation of different processes, including proliferation, differentiation, cell cycle regulation and apoptosis [8–15].

Despite the growing evidence accumulated on the antitumor effects of AdoMet in different cancer cells, [2, 8–15] poor data are presently available on the molecular mechanism exerted by the sulfonium compound.

Our previous studies showed that AdoMet exerts an inhibitory effect on the growth of breast cancer CG5 and MCF-7 cells [12–14]. In MCF-7 cells, AdoMet fulfills a strong inhibitory effect on cell proliferation by inducing both autophagy and apoptosis and in combination with chloroquine, an inhibitor of autophagy, potentiates apoptosis occurrence and AKT inactivation [12, 13]. In CG5 cells AdoMet increases apoptosis potentiating the effects of doxorubicin, one of the most used anticancer drug, by inducing activation of Fas/FasL pathway [14].

Recently, it has been demonstrated that the reduction in the expression of *MAT* genes in hepatocellular carcinoma can be attributed to the regulation of microRNAs (miRNAs), resulting in decreased AdoMet levels and deregulation of signal transduction pathways linked to methionine metabolism and MAT activity [16–18].

MiRNAs are a class of small non-coding 21–25 nucleotide single-stranded RNAs that regulate many physiological and pathological processes, like cell development, differentiation, infection, immunity, tumor suppression and carcinogenesis [19–21].

To date, at best of our knowledge, there are no works in literature that analyze the direct involvement of AdoMet in the modulation of non-coding RNAs levels.

The aim of this work was to get new insight into the molecular mechanisms underlying the antitumor activity of AdoMet through the study of the regulation of miRNAs expression profile in MCF-7 breast cancer cell line.

## Materials and methods

### Materials

AdoMet was provided from New England Biolabs, prepared in a solution of 5 mM H_2_SO_4_ and 10% ethanol, filtered and stored at 4 °C until use. Annexin V-fluorescein isothiocyanate (Annexin V-FITC) Apoptosis Detection kit was purchased from eBioscience (San Diego, CA). Monoclonal antibodies to caspase 9, caspase 8, Beclin1, p53, histone deacetylase 1 (HDAC1), AKT, pAKT, NAD-dependent deacetylase sirtuin-1 (SIRT1), poly (ADP-ribose) polymerase (PARP), phosphatase and tensin homolog (PTEN), β-actin, α-tubulin and polyclonal antibodies to ATG7, acetylated-p53^K382^ (Ac-p53), caspase 6, microtubule-associated protein light chain 3B (LC3B), were purchased from Cell Signaling Technology (Danvers, MA). Goat anti-rabbit IgGAlexa Fluor647 was provided from Abcam (Cambridge, UK). Horseradish peroxidase (HRP)—conjugated goat anti-mouse and goat anti-rabbit secondary antibodies were obtained from ImmunoReagents Inc. (Raleigh, NC). miRNA-34a, miRNA-34c and miRNA-486-5p mimics and inhibitors were obtained from Life Technologies (Waltham, MA). Lipofectamine 2000, mirVANA PARIS Kit, TaqManMiRNA Reverse Transcription Kit, Megaplex RT Primers, TaqManPreAmp Master Mix, MegaplexPreAmp Primers, TaqMan Universal PCR Master Mix, 384-well TaqManMiRNA Array CARD, Opti-minimal essential medium (Opti-MEM) and LysoTracker Red DND-99 (LTR), were obtained from Thermofisher Scientific (Massachusetts, USA).

### Cell cultures and transfections

The human breast cancer cell lines MCF-7 and MDA-MB 231 were obtained from the American Type Culture Collection (ATCC, Manassas, VA). Cells were cultured at 37 °C in a 5% CO_2_ humidified atmosphere and grown in Dulbecco’s modified Eagle’s medium (DMEM) supplemented with 10% heat-inactivated fetal bovine serum, 100 U/mL penicillin, 100 µg/mL streptomycin and 1% l-glutamine. Sub-confluent cells were seeded in 6-well plates at the density of 1.5 × 10^5^ cells/well to achieve 80% of confluence. After 24 h, cells were transfected with 100 nM miR-34a, miR-34c and miR-486-5p mimic or inhibitor, diluted in Opti-MEM free medium supplemented or not (Control) with 500 µM AdoMet, by using Lipofectamine 2000 according to manufacturer’s protocol. Lipofectamine was also used alone as a negative control. After 72 h from transfection, cells were harvested and then subjected to the extraction of the total RNA, preparation of cells lysates and flow cytometry analysis.

### MiRNA detection

Total RNA was isolated from cultured cells treated or not with AdoMet 500 µM, by using the mirVANA PARIS Kit, according to manufacturer instructions. Subsequently, using the TaqManMiRNA Reverse Transcription Kit and the Megaplex RT Primers, single-stranded cDNA was synthesized from total RNA samples. The selected cDNA targets were preamplified to increase the quantity of desired cDNA for gene expression analysis using TaqMan PreAmp Master Mix and the Megaplex PreAmp Primers. The preamplified cDNA targets were amplified by DNA polymerase from the TaqMan Universal PCR Master Mix using sequence-specific primers and probes on the 384-well TaqMan miRNA Array CARD. The array was loaded and run on Applied Biosystems Viia7 instrument (Life Techonologies, USA) by using the default thermal-cycling conditions.

### MiRNA validation by qRT-PCR

To validate the results of the Array CARDs, the expression of miRNAs was independently determined by quantitative real-time PCR (qRT-PCR). cDNAs were synthesized as detailed above and the expression of individual miRNAs was determined using pre-designed probe-primer sets from Life Technologies. To perform the qRT-PCR we used TaqMan miRNA Assays that use looped-primer RT-PCR to accurately detect mature miRNAs using Applied Biosystems ViiA7. During the target amplification step, the AmpliTaq Gold DNA polymerase amplifies target cDNA synthesized from the RNA sample, using sequence-specific primers from the TaqMan Assay Plates.

### Data analysis

Using comparative threshold cycle (Ct) method [22], we utilized endogenous controls to normalize the expression levels of target genes by correcting differences in the amount of cDNA loaded into qRT-PCR reactions. To normalize total RNA samples, the small-nuclear-U6 was selected as an appropriate constitutively expressed endogenous control.

### Flow cytometry analysis of apoptosis

Annexin V-FITC was used in conjunction with a vital dye propidium iodide (PI) to distinguish apoptotic (Annexin V-FITC-positive, PI positive) from necrotic (Annexin V-FITC negative, PI positive) cells [12, 23]. MCF-7 cells were plated in 6-multiwell plates at the density of 1.5 × 10^5^ cells/well and the day after, cells were transfected with 100 nM miR-34a and miR-34c mimic or inhibitor, with or without 500 µM AdoMet. After 72 h, cells were detached and analyzed as previously described [12].

### LysoTracker-red staining

MCF-7 cells were seeded in 6-well plates at the density of 1.5 × 10^5^ cells/well. After 24 h, cells were transfected with 100 nM miR-486-5p mimic or inhibitor, with or without 500 µM AdoMet. After 48 and 72 h, LTR was added for 20 min at 37 °C at a final concentration of 0.1 µM in DMEM. Cells were then washed with phosphate-buffered saline (PBS) and observed by fluorescence microscopy [24]. The fluorescence intensity was then analyzed by flow cytometry as previously described [12]. For the quantitative evaluation of LTR, FlowJo software was used to calculate median fluorescence intensities (MFI) by the formula (MFI-treated/MFI-control).

### LC3B detection

MCF-7 cells were seeded in 6-well plates at the density of 1.5 × 10^5^ cells/well. After 24 h, cells were transfected with 100 nM miR-486-5p mimic or inhibitor, with or without 500 µM AdoMet. After 72 h of treatment, cells were detached and analyzed as previously reported [12].

### Preparation of cell lysates

MCF-7 cells were transfected with 100 nM miR-34a, miR-34c and miR-486-5p mimic or inhibitor, treated or not with AdoMet 500 µM, and after 72 h, collected by centrifugation, washed twice with ice-cold PBS, and the pellet was lysed using 100 µL of RIPA buffer. After incubation on ice for 30 min, the samples were centrifuged at 18,000×*g* for 30 min a 4 °C, and the supernatant was recovered. Protein concentration was determined by Bradford method [25].

### Western blotting analysis

Equal amounts of cell proteins were separated by sodium dodecyl sulphate-polyacrylamide gel electrophoresis (SDS-PAGE) and electrotransferred to nitrocellulose membranes by Trans blot turbo (BIO-RAD). All primary antibodies were used at a dilution of 1:1000, while all secondary antibodies were used at a dilution of 1:5000. Blots were developed using enhanced chemoluminescence detection reagents ECL (Cyanagen, Bologna, IT) and exposed to X-ray film. All films were scanned by using Image J software (National Institutes of Health, USA).

### Statistical analysis

Experiments were performed at least three times with replicate samples, except where otherwise indicated. Data are expressed as mean ± standard deviation (SD). The means were compared using analysis of variance (ANOVA) plus Bonferroni’s t-test. A P-value of < 0.05 indicates a statistically significant result.

## Results

### MiRNA expression profile of AdoMet-treated MCF-7 cell line

Most of the studies regarding miRNA expression profiles have confirmed the existence of specific miRNAs in many types of neoplastic diseases, with the ability to regulate several genes in the context of the signaling pathways, involved in the promotion or suppression of carcinoma through regulation of gene expression at post-transcriptional levels [19–21].

**Fig. 1:**
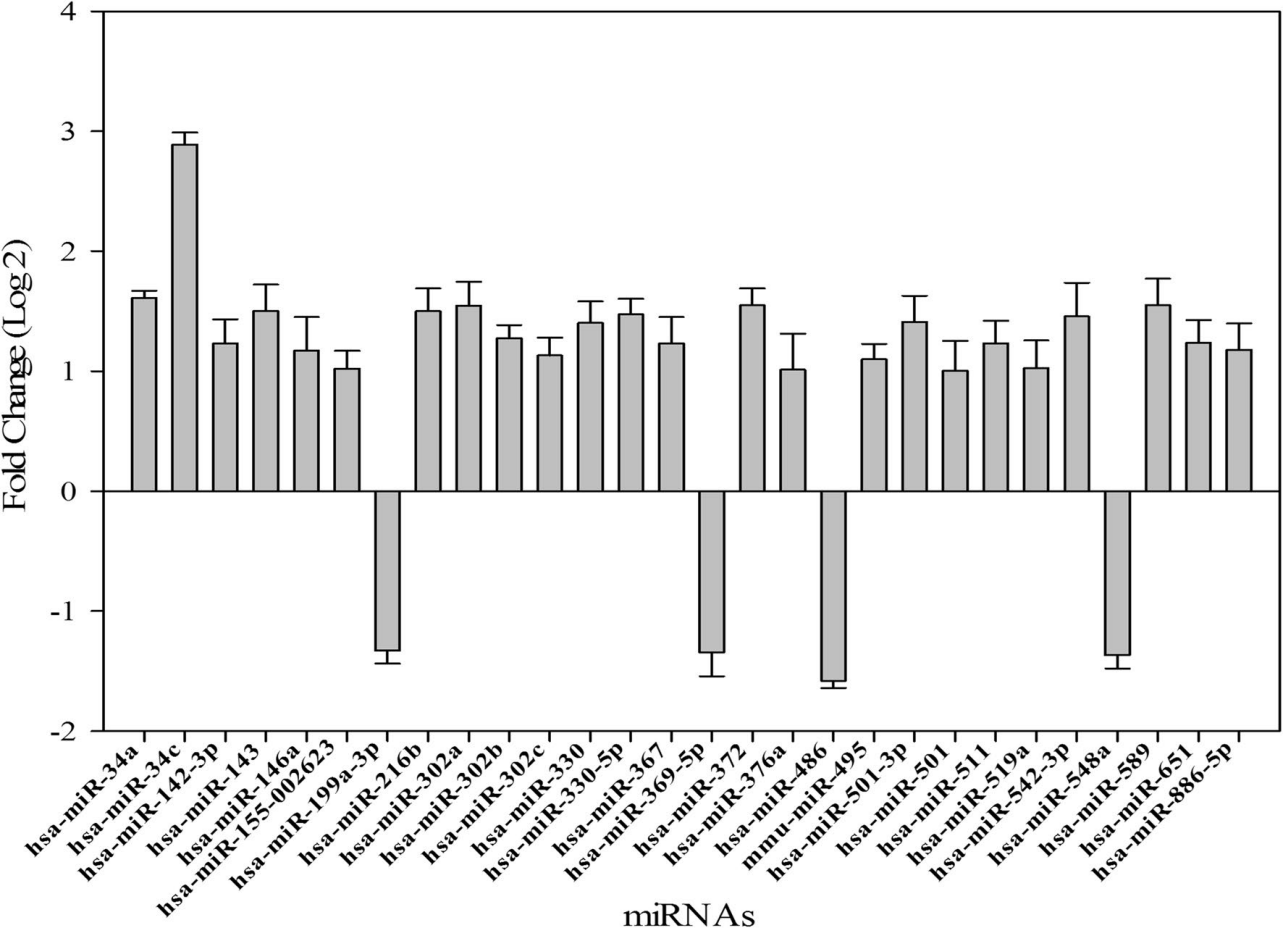
MiRNA expression pattern in AdoMet-treated MCF-7 cells. Cells treated with AdoMet 500 µM for 72 h, were subjected to miRNA expression profiling using a 384-well TaqMan Array CARD. Twenty-eight miRNAs were differentially expressed in AdoMet-treated cells if compared to control samples. Log2 fold change is graphically represented. The analysis was repeated at least three times and always gave similar results

In order to analyze the regulation of miRNA expression profiling following AdoMet-treatment in MCF-7 cells, we performed a miRNA expression profiling after 72 h of treatment with 500 μM AdoMet using a 384-well TaqMan Array CARD. Twenty-eight miRNAs were differentially expressed in AdoMet-treated cells if compared to control samples. The fold change is graphically represented in Fig. 1. Among them, we have focused the next investigations on those miRNAs that resulted the most regulated by AdoMet and that, at the same time, were the most involved in the regulation of the main pathways of proliferation and cell death, such as miR-34a, miR-34c and miR-486-5p.

As shown in Table 1, the validation of the results through the qRT-PCR analysis revealed that after 72 h of AdoMet treatment, when compared to untreated cells, miR-34a and miR-34c appeared remarkably up-regulated while the expression of miR-486-5p was down-regulated. These results were totally in agreement with the Array CARD data (Fig. 1).

**Table 1:** **Comparison of fold changes detected in microarrays and by qRT-PCR**

MiRNAs	Fold-change (Log2)			
	MCF-7		MDA-MB 231	
	Microarray	qRT-PCR	Microarray	qRT-PCR
hsa-miR-34a	1.61	1.42	N.E.	2.20
hsa-miR-34c	2.89	2.73	N.E.	2.25
hsa-miR486-5p	− 1.95	− 1.78	N.E.	3.25

*N.E.* not evaluated

In order to confirm the ability of the sulfonium compound to regulate miRNA expression in other breast cancer cells, we performed a qRT-PCR analysis after 72 h of 500 µM AdoMet treatment, in MDA-MB-231 breast cancer cell line, using pre-designed probe-primer sets for miR-34a, miR-34c, and miR-486-5p. The results obtained (Table 1) showed that, when compared to untreated cells, miR-34a and miR-34c appeared remarkably up-regulated (2.20- and 2.25-fold, respectively). These results, totally in agreement with those obtained on the MCF 7 cells, suggest a generalized regulation mechanism on miR-34a and miR-34c induced by AdoMet.

On the other hand, the expression of miR-486-5p resulted up-regulated of 3.25-fold compared to the control.

### MiR-34a and miR-34c enhance the pro-apoptotic effect of AdoMet in MCF-7 cells

**Fig. 2:**
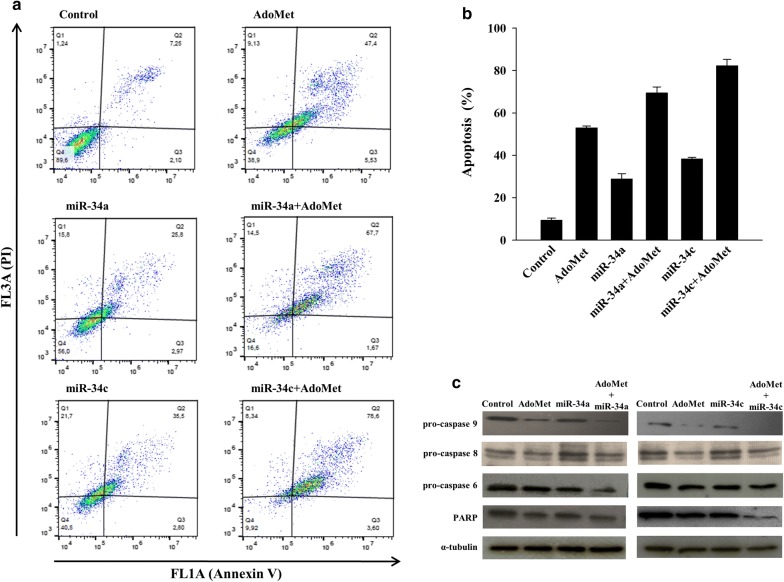
Effect of AdoMet, miR-34a and miR-34c mimic combination on apoptotic process in MCF-7 cells. Cells were not treated (control) or treated with 500 μM AdoMet and/or 100 nM miR-34a and miR-34c mimic for 72 h. **a** Apoptosis was evaluated by FACS analysis. The different quadrants report the percentage of cells: viable cells, lower left (Q4); early apoptotic cells, bottom right (Q3); late apoptotic cells, top right (Q2); and non-viable necrotic cells, upper left (Q1). The experiment was repeated three times and the results were always similar. **b** Quantification of apoptosis evaluated by the FACS analysis. The histogram plot shows the percentage apoptotic cells for single treatment. The analysis was the average of at least three independent experiments. **c** Western blot assay of MCF-7 cell extracts was evaluated for the expression of pro-caspase 9, pro-caspase 8, pro-caspase 6, and PARP. For the equal loading of protein in the lanes, the expression of the house-keeping protein α-tubulin was used as a standard. The images are representative of three immunoblotting analyses obtained from at least three independent experiments

To investigate the biological significance of miR-34a and miR-34c up-regulation in AdoMet-treated MCF-7 cells, we performed transfection experiments with either miR-34a or miR-34c mimic or with either miR-34a or miR-34c inhibitor and then the modulation of apoptosis was analyzed by flow cytometry after labeling the cells with Annexin V-FITC and PI 72 h after treatment. Flow cytometry analysis revealed that the transfection with 100 nM miR-34a and miR-34c induced about 29% and 38% apoptosis, respectively, while, in agreement with our previous results [12] about 50% of cell population underwent to apoptosis after treatment with AdoMet 500 μM (Fig. 2a, b). As shown in Fig. 2a, b, the combination of AdoMet and miR-34a or miR-34c mimic increased apoptotic cell death up to about 69 and 82%, respectively.

On the light of the pro-apoptotic effects induced by miR-34a or miR-34c mimic transfection combined with AdoMet treatment, we evaluated caspase activation cascade. MCF7 cells were transfected in presence or not of 500 μM AdoMet and caspases fragmentation was analyzed by Western blotting. As shown in Fig. 2c, AdoMet/miRNAs combined treatment strongly reduced the expression of full length caspase 9 and caspase 8, initiators of apoptosis, and of caspase-6, downstream caspase, with a concomitant decrease of full length PARP, a known target for apoptosis-associated caspase cleavage [26].

In conclusion, these data provide evidence that combination of AdoMet with miR-34a or miR-34c mimic potentiates the pro-apoptotic activity of AdoMet, by a caspase-dependent mechanism.

### MiR-34a and miR-34c modulate HDAC1 and SIRT1 expression

**Fig. 3:**
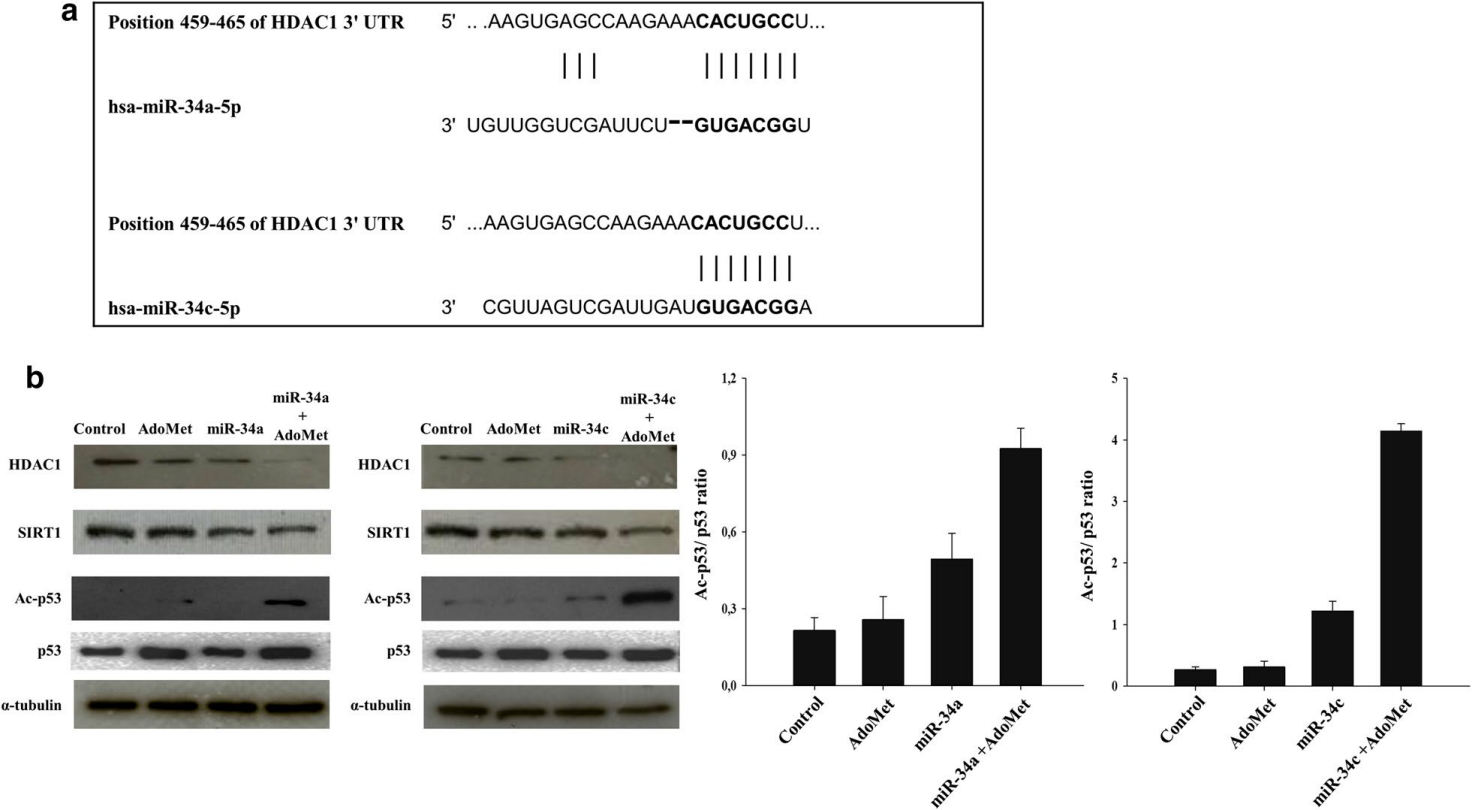
Effect of AdoMet and miR-34a mimic or miR-34c mimic on the HDAC1, SIRT1 and p53 expression levels. **a** Alignment of miR-34a and miR-34c with HDAC1 3′UTR obtained from miRNA-mRNA integration analysis using the TargetScan microRNA target prediction software. **b** Cells were transfected with miR-34a and miR-34c mimic or inhibitor, in the presence or not (Control) of 500 μMAdoMet for 72 h. Then, 10 μg of cell lysates were subjected to SDS-PAGE, incubated with antibodies against the indicated proteins and analyzed by Western blotting. The housekeeping protein α-tubulin was used as loading control. Graphs show the densitometric intensity of Ac-p53/p53 band ratio. The intensities of signals were expressed as arbitrary units. The images are representative of three immunoblotting analyses obtained from at least three independent experiments. Bars, SDs

To identify potential mRNA targets of miR-34a and miR-34c, we performed a miRNA-mRNA integration analysis by using the TargetScan miRNA target prediction software. The analysis identified the HDAC1 mRNA as potential target gene of miR-34a and miR-34c, and its binding sites for these miRNAs are shown in Fig. 3a. HDAC1 is an important epigenetic factor, which antagonizes the acetylation status of histone and non-histone proteins [27]. It is well known that HDAC1 is tightly correlate with cancer development and progression [28, 29] and it is reported that HDAC1 deacetylates p53, a critical master regulator in tumor suppression, thus modulating its effect on cell growth and apoptosis [30, 31]. In-depth literature studies have shown that miR-34 family holds the potential to regulate both SIRT1 protein expression and activity [32, 33].

SIRT1 is a nicotine adenine dinucleotide-dependent deacetylase that is involved in multiple biological processes, including DNA damage, apoptosis and proliferation. Several studies demonstrated that SIRT1 expression is correlated with tumor phenotype [34, 35] and was also able to deacetylate p53 [36, 37].

To confirm the interaction between miR-34a and miR-34c with HDAC1 and SIRT1, Western blotting analysis was performed to detect the expression of both proteins in MCF-7 cells after 72 h from transfection with both miRNA mimics.

Results showed that the HDAC1 and SIRT1 levels, as expected, were significantly reduced in MCF-7 transfected cells and that the combination of AdoMet with miR-34a or miR-34c mimic greatly enhanced the reduction of HDAC1 and SIRT1 expression (Fig. 3b). Surprisingly, the expression levels of both proteins resulted reduced also after AdoMet treatment alone.

These results were confirmed by the Western blot analysis of acetylated and deacetylated forms of p53. As shown in Fig. 3b, the Ac-p53 protein expression levels were significantly increased by the up-regulation of miR-34a or miR-34c AdoMet-mediated and/or trasfection-induced, as indicated by the significant increase of Ac-p53/p53 ratio thus providing an indirect evidence that AdoMet in combination with miR-34a or miR-34c was able to strongly decrease both HDAC1 and SIRT1 expression.

The obtained results showed that AdoMet in combination with miR-34a or miR-34c mimic was able to strongly decrease both HDAC1 and SIRT1 protein levels.

### MiR-486-5p inhibitor enhances the pro-autophagic effect of AdoMet in MCF-7 cells

We previously demonstrated that AdoMet strongly induces the activation of autophagy, after 48 and 72 h of treatment at the dose of 500 µM [12].

**Fig. 4:**
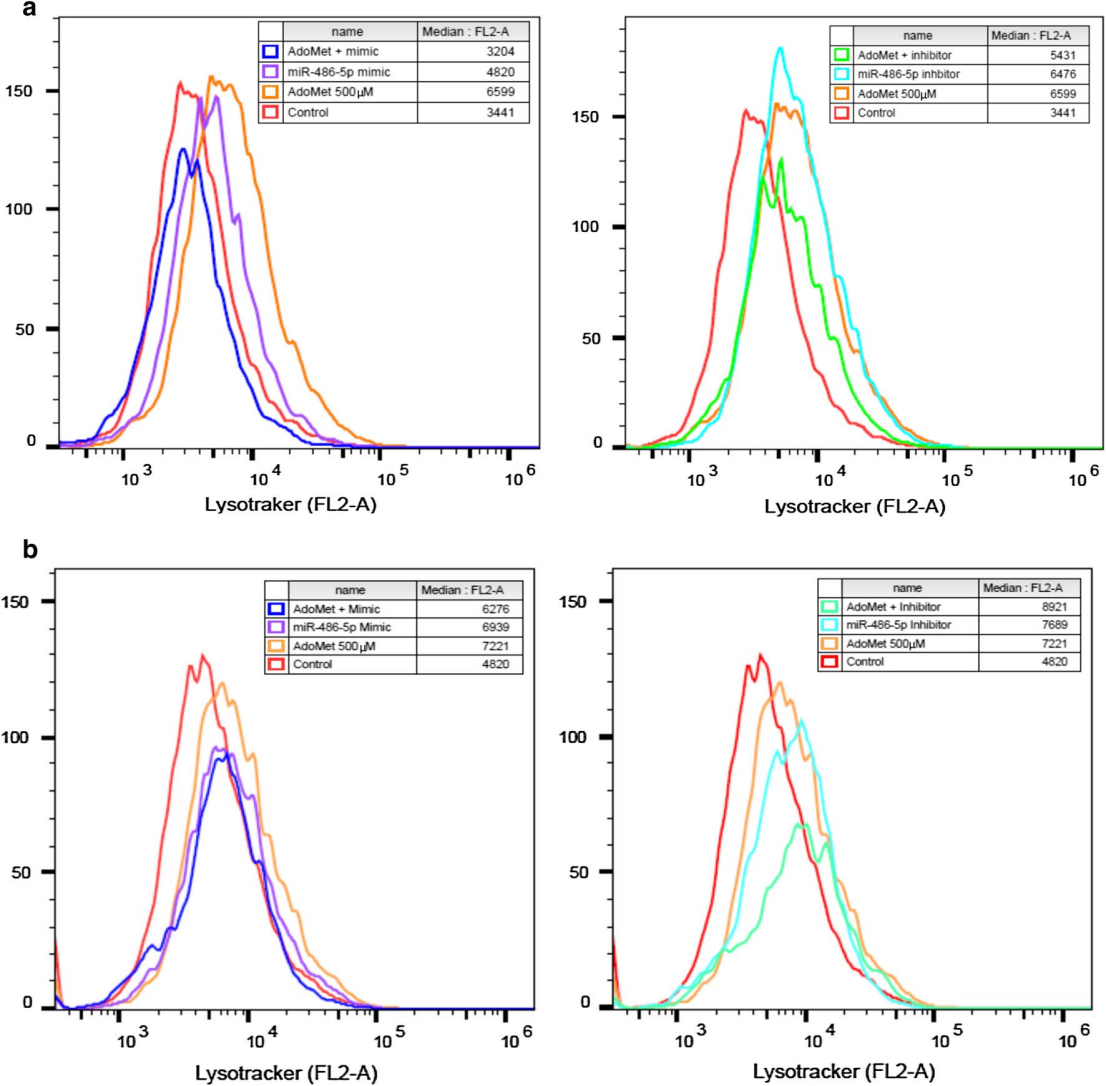
Autophagy occurrence in MCF-7 after AdoMet and miR-486-5p mimic or inhibitor treatment. Cells were not treated (Control) or treated with 500 μM AdoMet and/or transfected with miR486-5p mimic or miR-486-5p inhibitor for 48 and 72 h. MCF-7 cells were exposed to LTR and analyzed by flow cytometry. **a** Autophagy flux evaluated after 48 h of AdoMet treatment. **b** Autophagy flux evaluated after 72 h of AdoMet treatment. The experiments were repeated at least three times and always gave similar results. The percentage of MFI was calculated as % of untreated control. Values are the means of three independent experiments.

To investigate the possible implication of miR-486-5p in the autophagic induction by AdoMet, cells were transfected for 48 and 72 h with both miR-486-5p mimic or inhibitor, in presence or not of AdoMet. The evaluation of the autophagic flux was performed by flow cytometry after cell-staining with LTR, a fluorescent probe for labeling and tracking of acidic organelles in living cells [23]. The flow cytometry analysis showed that after 48 h of the treatment (Fig. 4a) miR-486-5p inhibitor and AdoMet were able to increase the autophagic flux respect to the control cells, but the combination of AdoMet with miR-486-5p inhibitor did not improve the effects obtained with the two molecules alone. Figure 4b showed that after 72 h of treatment miR-486-5p mimic and inhibitor alone are both able to increase the autophagic flux (on the left and on the right of the figure, respectively), and in particular miR-486-5p inhibitor caused a greater effect than that obtained with AdoMet. The combination of AdoMet and miR-486-5p mimic determined a decrease of the effects obtained with the two molecules alone, while the combination of AdoMet with miR-486-5p inhibitor strongly potentiated the effect obtained with AdoMet alone (Fig. 4b).

**Fig. 5:**
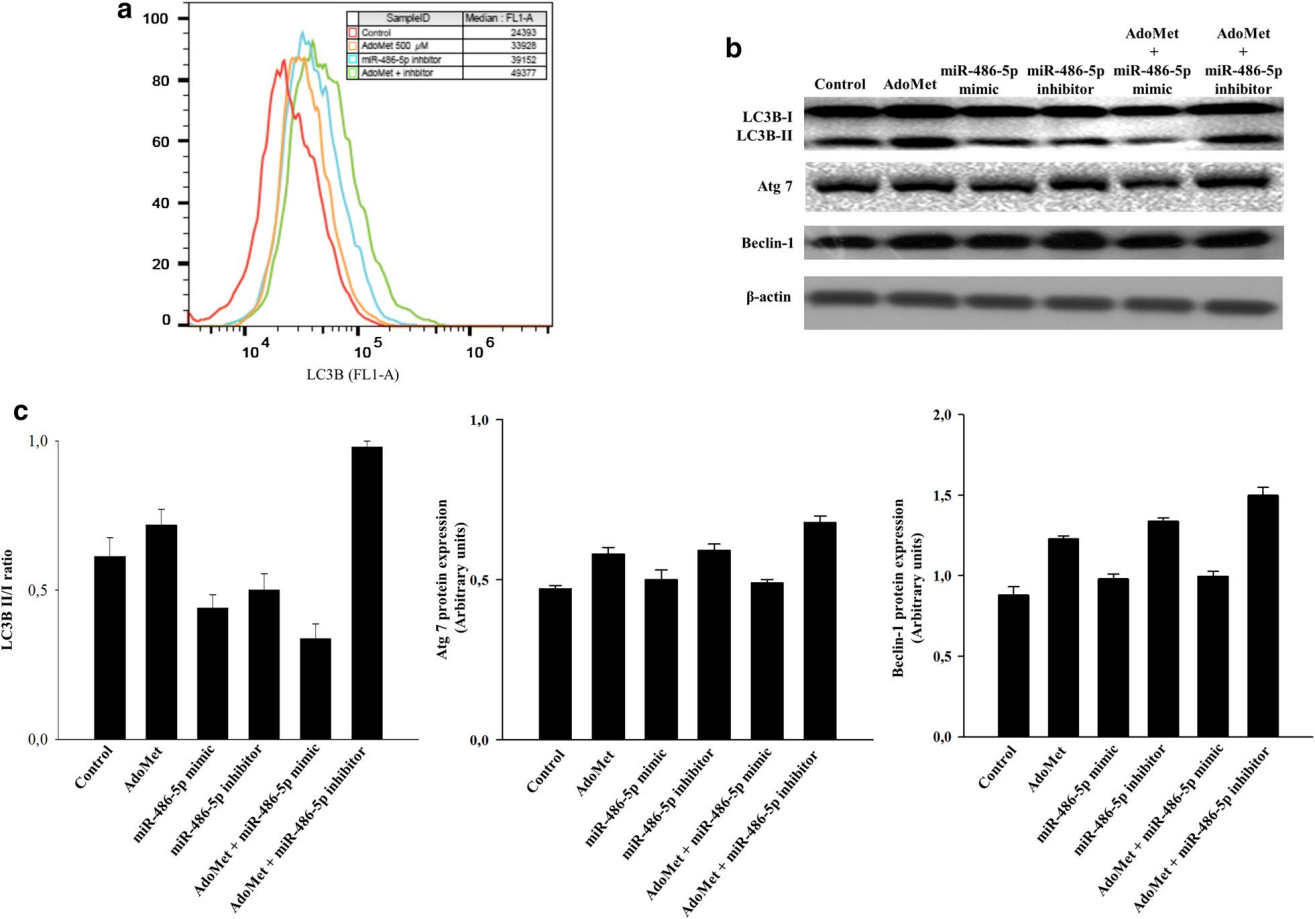
Effect of AdoMet and miR-486-5p mimic or inhibitor combination on the autophagy process. Cells were not treated (Control) or treated with 500 μM AdoMet and/or transfected with miR-486-5p mimic or miR-486-5p inhibitor for 72 h. **a** Cells were labeled with anti-LC3B antibody and then analyzed by flow cytometry after staining with goat anti-rabbit IgG (Alexa Fluor647). The percentage of MFI was calculated as % of untreated control. Values are the means of three independent experiments. **b** Western blot assay for the expression of LC3B, Beclin1 and ATG7. **c** Graphs show the densitometric intensity of Beclin 1 and ATG7 bands, and LC3BII/I bands ratio. The intensities of signals were expressed as arbitrary units. For the equal loading of protein in the lanes, β-actin was used. Bars, SDs

To confirm the results obtained, we detected the level of LC3B after staining with anti-LC3B polyclonal antibody. Flow cytometry analysis performed after 72 h of treatment revealed that the inhibition of miR-486-5p led to a significant increase in LC3B signals, compared to control cells, and that this effect was potentiated by the combination with AdoMet (Fig. 5a).

To confirm the data obtained by flow cytometry, we performed Western blotting analysis in order to evaluate the level of the autophagy-related markers ATG7, Beclin1 and LC3B. ATG7 functions as an E1 enzyme essential for  different substrates, needed for the autophagic vacuole formation [38]. Beclin 1, the mammalian orthologue of yeast Atg 6, is a key regulatory protein in the autophagic pathway that participates to the assembly of the autophagy-initiation complex [39, 40]. LC3B is a protein normally located in the cytosol (LC3B-I) but upon induction of autophagy it becomes lipidated, cleaved to form LC3B-II, and embedded in autophagosomal membranes [41, 42]. Thus, the amount of LC3B-II is correlated with the extent of autophagosome formation. As shown in Fig. 5b, c, after 72 h of treatment the combination of AdoMet and miR-486-5p inhibitor  augmented the LC3B-II/LC3B-I ratio by increasing the levels of LC3B-II, according to the data obtained through FACS analysis, as well as enhanced the levels of ATG7 and Beclin1.

Taken together, these data provide evidences that the inhibition of miR-486-5p can potentiate the pro-autophagic activity of AdoMet in MCF-7 cells.

### PTEN is a potential target of miR-486-5p

**Fig. 6:**
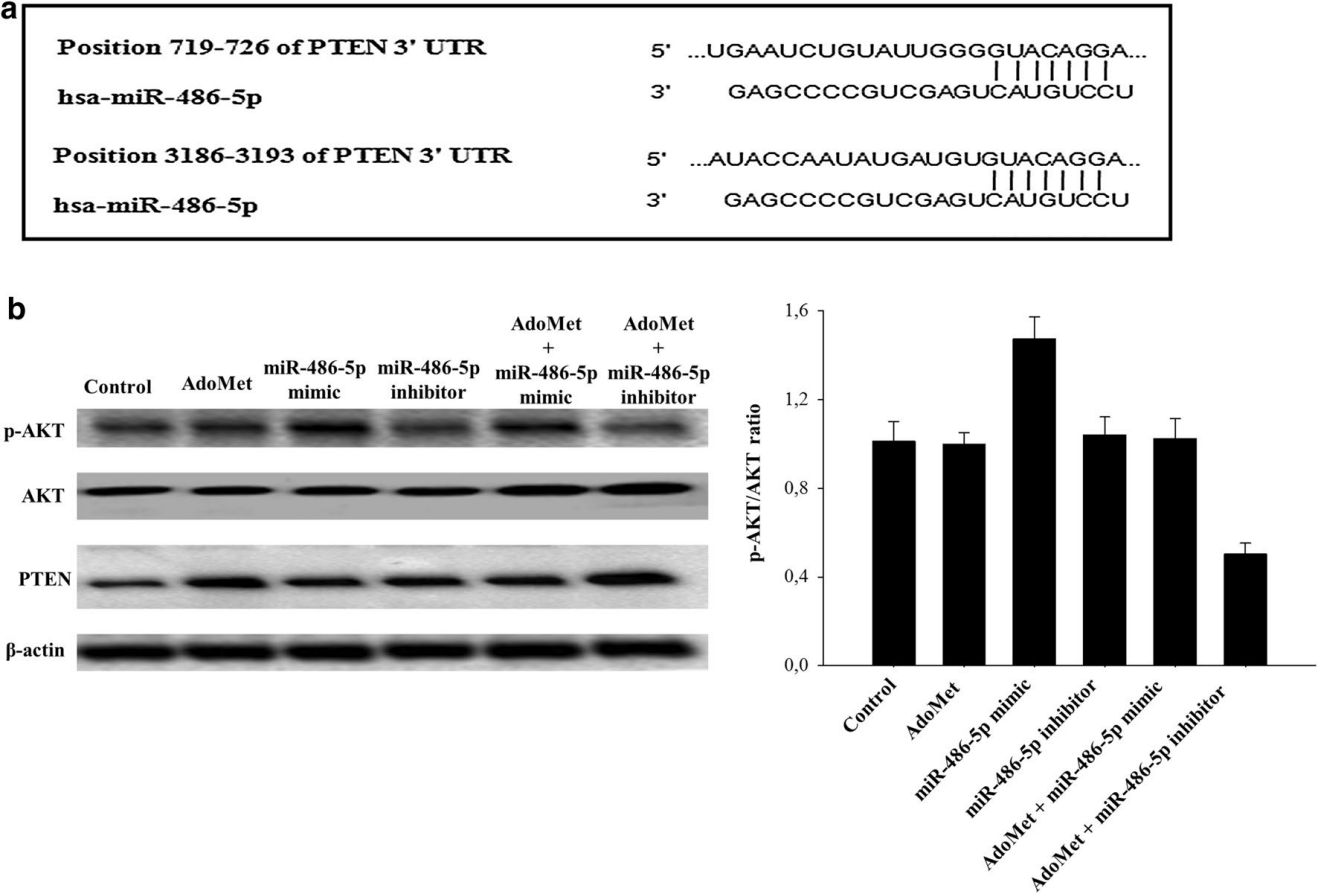
Effect of AdoMet and miR-486-5p mimic or inhibitor on PTEN/AKT signaling pathway. **a** Alignment of miR-486-5p with PTEN 3′UTR obtained from miRNA-mRNA integration analysis using the TargetScan microRNA target prediction software. **b** Cells were transfected with miR-486-5p mimic or inhibitor, in the presence or not (Control) of 500 μMAdoMet for 48 and 72 h. Then, 10 μg of cell lysates were subjected to SDS-PAGE, incubated with antibodies against the indicated proteins and analyzed by Western blotting. The housekeeping protein β-actin was used as loading control. Graphs show the densitometric intensity of pAKT/AKT bands ratio. The intensities of signals were expressed as arbitrary units. Bars, SDs

To identify potential mRNA targets of miR-486-5p, we performed a miRNA-mRNA integration analysis by using the TargetScan microRNA target prediction software. The analysis identified 168 potential targets, based on their sequence complementarity. Among them, we focused our attention on PTEN, which is an important component of the phosphatidylinositol 3-kinase (PI3K)/AKT signaling pathway [43]. Figure 6a shows the predicted based-pairing between miRNA-486-5p and the 3′-UTR of PTEN mRNA.

To test directly the potential involvement of miR-486-5p in the regulation of PI3K/AKT signaling pathway, MCF-7 cells were transfected with miRNA-486-5p mimic or inhibitor, diluted in free medium or in medium supplemented with 500 µM AdoMet. After 72 h from transfection, total protein content was analyzed by Western blotting. Figure 6b shows that the combination of AdoMet and miR-486-5p inhibitor increased PTEN protein levels and decreased AKT phosphorylation. Notably, in the same time, the expression levels of PTEN after AdoMet treatment alone resulted up-regulated. Interestingly, miR-486-5p mimic reduced the effects of AdoMet on the increased expression of PTEN, while increased the phosphorylation status of AKT as shown by pAKT/AKT ratio.

## Discussion

Breast cancer is one of the most frequently diagnosed diseases and one of the leading causes of cancer death in women despite significant progress in both diagnosis and therapy [44–46]. The high incidence of breast cancer in developing countries and its poor prognosis partially attributed to multiple-drug resistance and anti-apoptosis activity of cancer cells has prompted scientists to discover more effective and less toxic therapeutic and preventive strategies [47–49].

The potential of AdoMet as antiproliferative agent has been evidenced in the literature, and growing scientific interest is focused on identifying the biological mechanisms and the signal transduction pathways related to the chemo-preventive activity of this physiological compound. AdoMet, due to its ability to methylate and, consequently, to silence pro-metastatic genes, is able to affect tumor progression, invasiveness and metastasis formation. Our previous study showed that AdoMet synergistically potentiates the antitumor effect of doxorubicin in the regulation of breast cancer CG5 cell proliferation [14]. Moreover, AdoMet in combination with chloroquine modulates the process of autophagy that represents an important mechanism of escape from apoptosis thus providing the possibility to improve the pharmacological therapy of breast cancer [12, 13].

In recent years, a new class of small non-coding RNA molecules, known as miRNAs has been associated with several human diseases including breast cancer. MiRNAs are emerging as potential diagnostic, prognostic and therapeutic tools for cancer treatment and it is believed that modulating miRNA expression may represent a potential therapeutic strategy for treating cancer.

MiRNAs act as post-transcriptional regulators through binding the 3′UTR regions and influencing the expression of targets [19–21]. Several studies have demonstrated that miRNAs can act as potential oncogenes or as tumor suppressor genes during the progression of cancer, as well as crucial regulators in carcinogenesis and tumor progression [19–21]. Recent studies reported the capability of miRNAs to regulate the expression of *MAT* genes, modulating AdoMet cell levels [16–18].

To date, a direct correlation between the antiproliferative effect of AdoMet and the variation of miRNAs expression has never been shown.

In the current study, we demonstrate for the first time that AdoMet was able to modify miRNAs expression profile in MCF-7 and MDA-MB 231 breast cancer cell lines. AdoMet treatment significantly modulated three miRNAs, up-regulating miR-34a and miR-34c in both cell lines and down-regulating and up-regulating miR-486-5p expression, in MCF-7 and MDA-MB 231 cells, respectively.

The miR-34 family consists of three members, miR-34a, miR-34b, and miR-34c [32, 33]. Their promoter region has a p53 binding site and, therefore, they are induced by p53 and involved in cell growth inhibition and apoptosis [32, 33].

It has been widely studied that miR-34 is involved in the control of cancer growth by targeting different tumour-related genes, and for this reason it could be considered a predictive biomarker in cancer [32, 33]. A decreased expression of miR-34 has been found in numerous malignancies [50–55]. Studies on miR-34a expression in breast cancer have shown that miR-34a is significantly down-regulated in about 32% and highly expressed in about 25% of the tumours.

Regarding miR-34c, Achari and colleagues demonstrated that miR-34c exert tumour-suppressive effects in breast cancer through different mechanisms, such as induction of G2/M cell cycle arrest and suppression of the pro-survival factors BCL2 or SIRT1 [56]. Overall, there are several ongoing studies aimed to better understand the avail of the miR-34 replacement, in order to improve the clinical outcome of cancer patients.

To investigate the effects of miRNAs deregulation in AdoMet-treated MCF-7 cells, we transfected cells with miR-34a and miR-34c mimics or inhibitors and evaluated the changes in the main antiproliferative signaling pathways and cell death processes by flow cytometry and Western blotting.

We demonstrated that the combination of AdoMet with either miR-34a or miR-34c mimic potentiated the pro-apoptotic activity of AdoMet, by a caspase-dependent mechanism. To further elucidate the molecular mechanisms of miR-34a and miR-34c, we sought to identify their putative target genes. Through the bioinformatic TargetScan software we identified potential miRNA-mRNA-protein interactions and among the 633 candidate targets we focused our attention on HDAC1.

HDAC1 over-expression is related to the development of some cancers [27–29]. Sun and colleagues demonstrated that miR-34a expression is negatively associated with HDAC1, and that miR-34a may act as a tumour suppressor gene regulating HDAC1 expression and inducing cell cycle arrest and apoptosis in HCC [57]. Furthermore, Yamakuchi and colleagues provide evidences that SIRT1 can be recognized and targeted by miR-34a and miR-34c in colon cancer cells. It has been showed that the inhibition of SIRT1 mediated by miR-34 leads to an increase of p53 acetylation, thus enhancing its stability and activity [58, 59].

Here we demonstrated that the combination of AdoMet and either miR-34a or miR-34c repressed SIRT1 and HDAC1 expression, paralleled by the increase of p53 acetylation. We also showed that the combined treatment with AdoMet and miR-34a or miR-34c greatly potentiates the AdoMet-induced apoptosis in MCF-7 cells. The increase of p53 acetylated status likely caused an enhancement of its stability [60], leading to the enhancement of apoptosis induced by AdoMet.

While miR-34 family has been extensively studied and its correlation with cancer as well as its molecular targets have been well characterized, there is still much to investigate about miR-486.

It is known that miRNA-486-5p plays an important role in various types of cancers. MiR-486-5p is one of the most down-regulated miRNAs in lung tumor tissues and contributes to lung cancer progression and metastasis, while its presence in the sputum and plasma specimens could provide a diagnostic approach for the early detection of lung cancer [61, 62]. Furthermore, it causes the reduction in the phosphorylation and activation of AKT and its downstream phospho-forkhead box 03A [63].

MiR-486-5p has been found to be significantly down-regulated in primary gastric cancer (GC) and in GC cell lines, and genomic loss of the miR-486 locus has been shown in approximately 25–30% of GC, which is consistent with its tumor-suppressive role [64]. Moreover, it has been reported that in esophageal squamous carcinoma cells a down-regulation of miR-486-5p acts as a tumor suppressor [65] while in prostate cancer it has been proposed that a significant over-expression of miR-486-5p contributes to the onset of the tumor phenotype through negative regulation of multiple tumor suppressor pathways [66]. Deregulation of miR-486-5p and its role as tumor suppressor has also been reported in breast cancer [67]. Despite the extensive literature the function and clinical significance of miR-486-5p in cancer remains controversial.

Here we demonstrated that miR-486-5p inhibitor led to increased autophagy and potentiated the pro-autophagic effect of AdoMet. This result was paralleled by an increase in protein levels of the autophagic markers LC3B-II, Beclin1 and ATG7, analyzed by Western blotting. As expected the combination with miRNA mimic led to a decrease in the pro-autophagic effect of AdoMet.

In order to clarify the molecular mechanism of autophagy induction mediated by miR-486-5p, we identified by TargetScan its putative target genes. One-hundred-sixty-eight targets were identified and, among them, PTEN, a tumor suppressor which negatively affects the PI3K-AKT signaling pathway, acting as a potent inhibitor of growth and survival signaling has been selected. PTEN directly dephosphorylates and thereby inhibits PI3K, which in turn suppresses AKT activity and allows the induction of autophagy. PTEN gene is located on chromosome 10q23, which is often deleted or mutated in various cancers resulting in the constitutive activation of AKT pathway, and, therefore, in the inhibition of autophagy.

Our findings showed that the combination of AdoMet and miR-486-5p inhibitor strongly increased the levels of PTEN, reduced AKT phosphorylation and potentiated the proautophagic effect of AdoMet. Therefore, miR-486-5p could be considered a novel direct modulator of AdoMet-dependent autophagy induction in MCF-7 cells.

## Conclusions

Collectively, our results provide the first evidence that AdoMet regulates miRNA expression in MCF-7 cells and give new insights into the mechanism by which the sulfonium compound exerts its antitumor effect suggesting the use of AdoMet as an attractive miRNA-mediated chemopreventive and therapeutic strategy in breast cancer.
